# Idiopathic Regression in Down Syndrome

**DOI:** 10.1192/bjo.2025.10704

**Published:** 2025-06-20

**Authors:** Ayomipo Amiola, Manilka Brahmana, Ignatius Gunaratna, Regi Alexander

**Affiliations:** 1 Hertfordshire Partnership University NHS Foundation Trust, Little Plumstead, United Kingdom; 2 University of Hertfordshire, Hatfield, United Kingdom

## Abstract

**Aims:** Idiopathic Regression in Down Syndrome (IRDS) is reported to be present in 16% of people with Down syndrome however the clinical presentation is heterogeneous with no universal diagnostic criteria. It often presents in adolescence or early adulthood and there are often no known triggers. Common symptoms include language regression, mood symptoms, psychotic phenomena, motor symptoms and loss of previously acquired cognitive skills.

We present a case series of two patients who presented to the West Norfolk Community Intellectual Disability Service with symptoms suggesting IRDS.

**Methods:** AB (F; 34 years) has the diagnoses of Mild Learning Disability, Down syndrome, Bipolar Affective Disorder following a manic episode at the age of 18 and obsessive-compulsive disorder with predominantly compulsive acts. She was described by parents as a very sociable, active, and high achieving before she developed acute regression. Around the age of 12 years following an episode of profoundly serious pneumonia, she became catatonic, anorexic, and doubly incontinent. Clearly described episodes of depression and mania, obsessional behaviours and speech deterioration were also noted.

The diagnosis of IRDS was raised by parents in 2023 and AB is currently being assessed for immunotherapy.

XY (M; 46 years). Following a gastrointestinal infection aged 18 years, the family noticed he became more housebound, obsessional about symmetry, and depressed. No specialist investigations were done but he was managed for depression with several antidepressants with no improvement. He was also diagnosed with dementia and started on donepezil but nothing changed. He is currently psychotropics-free and following a retrospective diagnosis of IRDS and discussion with family, they were relieved that the correct diagnosis of XY’s condition has been found.

**Results:** A physical illness appears to have triggered the regression in both cases. Personality and mood changes especially a manic presentation which is uncommon in people with Down syndrome were also reported. Psychotropic medications were not beneficial in at least the second case. In both cases, the diagnosis of Idiopathic Regression in Down Syndrome was an acceptable explanatory model for the family.

**Conclusion:** We hope clinicians will make the diagnosis more promptly thus facilitating quick access to adequate treatment.

